# Functional and anatomical alterations in bilateral vestibulopathy: A multimodal neuroimaging study and clinical correlation

**DOI:** 10.3389/fneur.2023.1157931

**Published:** 2023-03-30

**Authors:** Eek-Sung Lee, Young Cheol Weon, Ji-Soo Kim, Tae-Kyeong Lee, Ji-Yun Park

**Affiliations:** ^1^Department of Neurology, Soonchunhyang University Bucheon Hospital, Bucheon, Republic of Korea; ^2^Department of Radiology, University of Ulsan College of Medicine, Ulsan University Hospital, Ulsan, Republic of Korea; ^3^Department of Neurology, Seoul National University Bundang Hospital, Seoul National University School of Medicine, Seoul, Republic of Korea; ^4^Department of Neurology, University of Ulsan College of Medicine, Ulsan University Hospital, Ulsan, Republic of Korea

**Keywords:** bilateral vestibulopathy (BV), head impulse test (HIT), dynamic visual acuity (DVA), vestibulo-ocular reflex (VOR), cognition, spatial orientation

## Abstract

**Object:**

To study multimodal neuroimaging study including resting state functional MRI (rs-fMRI), anatomical connectivity and brain morphology in patients with bilateral vestibulopathy (BVP) and relationship with clinical correlation.

**Methods:**

Thirteen patients with BVP (7 women; mean age ± SD = 63.5 ± 14.7 years, 22–80 years) and eighteen age and gender-matched controls were compared rs-fMRI and anatomical MRI. Also, we analyzed the relationship between multimodal neuroimaging and Dizziness Handicap Inventory score (DHI), Vestibular Disorders Activities of Daily Living Scale (VDRL), Geriatric Depression Scale (GDS) and Hospital Anxiety and Depression Scale (HADS).

**Results:**

Compared with controls, BVP patients showed decreased functional connectivity among the key nodes of the salience network, auditory (including vestibular) network, bilateral posterior parahippocampal gyri, bilateral paracingulate gyri, and right frontoparietal network, and the anatomical connectivity in the right cerebellum, corpus callosum tapetum, and left fornix. BVP patients showed decreased gray matter volume in the bilateral parahippocampal gyri, right precentral gyrus, anterior cingulate gyrus, and right middle temporal gyrus and increased gray matter volume in the right superior frontal gyrus compared with controls. Correlation analyses showed rs-fMRI and clinical variables showed no significant result. DHI correlated negatively with anatomical connectivity in the bilateral frontal parahippocampal cingulum, corpus callosum, right inferior fronto-occipital fasciculus, bilateral fornix, and gray matter volumes in the bilateral middle occipital gyri, right superior occipital gyrus, left angular gyrus, and right cuneus in BVP. VADL correlated negatively with Anatomical connectivity in the corpus callosum, bilateral fornix, bilateral cerebellum, bilateral superior and anterior thalamic radiation, right inferior fronto-occipital fasciculus, bilateral fronto-parietal cingulum, right dentatoruburothalamic tract and gray matter volumes in the right angular gyri, bilateral parahippocampal gyri, right middle temporal gyrus, right cuneus, bilateral inferior occipital gyri, left middle occipital gyrus, right superior frontal gyrus, left fusiform gyrus, bilateral caudate, left cerebellar crus, and bilateral calcarine gyri in BVP.

**Conclusions:**

This study identified reductions in the volume of the hippocampus and alterations in functional and anatomical connectivity that concurs with previously established characteristics of BVP. The degree of disability can be inferred from the change in the connectivity and volume between vestibular cortical areas and their network.

## Introduction

Bilateral vestibulopathy (BVP) is characterized by chronic dizziness, unsteadiness and oscillopsia due to impaired or loss of function of the peripheral vestibular system bilaterally (1–3). There were diverse high cortical dysfunctions beyond leading symptoms due to direct vestibular deficits.

Several studies have established that patients with BVP exhibit impairments in attention and executive function (4–6), as well as spatial cognition, including spatial memory and navigation (5–10). Furthermore, structural and functional brain imaging studies have reported hippocampal atrophy (8), decreased bilateral activation in the parieto-insular vestibular cortex (PICV) during caloric stimulation (11), and altered connectivity patterns in the posterior insula, parietal operculum, and cerebellum in resting-state functional MRI (rs-fMRI) (12).

However, previous studies have several limitations. The diagnostic criteria for previous studies were quite variable across studies from complete vestibular loss to mild vestibular loss. In 2017, the Classification Committee of the Barany Society describes the diagnostic criteria for BVP that is most commonly used for the diagnosis of BVP currently (13). Therefore, we enrolled BVP patients who meet the Barany society criteria. Additionally, it is imperative to employ data-driven analysis, as previous studies have been restricted to investigating particular regions of the brain such as the hippocampus, or have solely focused on examining brain responses to specific tasks. Our multimodal neuroimaging study analyzed brain morphology and functional and anatomical connectivity in patients with BVP to investigate disease-related brain changes using voxel-based morphometry, functional connectivity analyses, and tractography analysis.

## Materials and methods

### Participants

Thirteen patients with BVP (7 women; mean age ± SD = 63.5 ± 14.7 years, 22–80 years) were enrolled at the Dizziness Clinic at Ulsan University Hospital between July 2018 and July 2022. All patients and controls underwent the Mini-mental state examination (MMSE) to rule out the dementia and patients with a score of 24 or higher were included. Also, they did not complain of hearing disturbance, and all patients with BVP showed no specific findings in pure tone audiometry. BVP patients were compared with age and gender-matched eighteen healthy subjects served as controls (10 women; mean age ± SD = 57.1 ± 10.51 years, range 36–74 years, *p* = 0.171; MMSE score = 29.82 ± 0.39, range 29–30, *p* = 0.004). Table 1 presents the diagnostic criteria of BVP proposed by the Classification Committee of the Bárány Society. Bárány's criteria proposed only the criteria for water stimulation in the caloric test, but since we performed the caloric test with air stimulation instead of water stimulation, we present the diagnostic criteria suitable for our laboratory (13). Patients with neurological or psychiatric disorders that may affect resting-state brain activities, previous history of head trauma, dementia (MMSE score ≤ 23), hearing losss, history of generalized anxiety disorder or major depressive disorder, or medication that may affect cerebral function, such as antidepressants or anxiolytics were excluded from the study. All subjects were right-handed according to the Edinburgh Handedness Inventory (14) and had completed Korean versions of the Dizziness Handicap Inventory (DHI, range: 0–100) (15), Geriatric Depression Scale (GDS) and the Hospital anxiety and depression scale (HADS, range: 0–42) (16). The study was followed by the Declaration of Helsinki and approved by the Institutional Review Board of Ulsan University Hospital (IRB no. 2018-06-016).

**Table 1:** Diagnostic criteria for bilateral vestibulopathy according to the classification Committee of the Bárány Society (13).

A. Chronic vestibular syndrome with the following symptoms 1. Unsteadiness when walking or standing1 plus at least one of 2 or 3 2. Movement-induced blurred vision or oscillopsia during walking or quick head/body movements and/or 3. Worsening of unsteadiness in darkness and/or on uneven ground
B. No symptoms while sitting or lying down under static conditions
C. Bilaterally reduced or absent angular VOR function documented by 1. Bilaterally pathological horizontal angular VOR gain < 0.6, measured by the video-HIT or scleral-coil technique and/or 2. Reduced caloric response (sum of bithermal max. peak SPV on each side < 6°/s) and/or 3. Reduced horizontal angular VOR gain < 0.1 upon sinusoidal stimulation on a rotatory chair (0.1 Hz, Vmax = 50°/s) and a phase lead >68 degrees (time constant < 5 s).
D. Not better accounted for by another disease

### Video-head impulse test (vHIT)

We used a video-head impulse tests (vHIT) device (SLMed, Seoul, Korea) to evaluate horizontal semicircular canal functions. Patients were seated upright with 30° of head flexion. A single right-handed examiner conducted head impulses by rotating the patient's head to the right and left while the patient fixated on a stationary target on the wall at a distance of 1 m. Head impulses were applied with a peak velocity range of 200–250°/s, rotation amplitude of 15–20°, and duration of 150–200 ms. A minimum of 20 horizontal head impulses were delivered randomly in the right or left direction. The software automatically calculated the gain in VOR as a ratio of angular eye velocity to angular head velocity or area under the curve (AUC) of the eye velocity to AUC of the head velocity. The vHIT was defined as bilaterally pathological if each side VOR gains were reduced below a value of 0.6 and overt and/or covert correcting saccades were recorded like Bárány's criteria.

### Caloric test with air stimulation

In our study, a bi-thermal caloric test with air stimulation was performed with a binocular video oculography system (SL Med, Seoul, Korea) with the patient in a supine position with 30° of head flexion. For the caloric test, an airflow was presented for 60 s, with a flow of 8 L/min, at temperatures of 50°C and 24°C. This air stimulation generated an endolymphatic current similar to that generated with water at temperatures of 44°C and 30°C in the caloric test (17). In our lab, the normal range of the sum of bithermal maximal peak slow-phase velocity on the left side is 24.79 ± 9.82°/s and right 30.07 ± 12.00°/s. we set the sum of bithermal max. peak SPV on each side < 6°/s as reduced or absent caloric response bilaterally like Bárány's criteria.

### Imaging acquisition

All subjects had MRIs using a 3T Philips Achieva MRI scanner (Philips Healthcare, Inc., Best Netherlands) at Ulsan University Hospital. Subjects were asked to rest with their eyes closed and lie still during the scanning. High-resolution three-dimensional anatomic T1-weighted images of the brain were acquired with 0.5-mm isotropic voxels using a fast field-echo planar imaging sequence (TR = 8.1 ms, TE = 4.6 ms, flip angle = 8°, slice thickness = 1 mm, 175 slices). Functional images of the whole brain were acquired using a fast field-echo planar imaging sequence (TR = 3,000 ms, TE = 30 ms, matrix = 64 × 64, FOV = 224 mm, flip angle = 90°, slice thickness = 3.5 mm/0 gap, numbers of slice = 42, number of volumes = 120, and total scan time = 6 min 9 s).

### Functional connectivity analysis

A total of 31 diffusion MRI scans (13 BVP subjects, 18 control subjects) were included. The images were preprocessed using the CONN toolbox version 20b (http://www.nitrc.org/projects/conn), which was implemented in SPM version 12 (http://www.fil.ion.ucl.ac.uk/spm/software/spm12) working on MATLAB 2016a (MathWorks, Inc., Natick, MA, USA). The preprocessing steps were applied with default parameters in CONN as follows: The preprocessing steps were applied with default parameters in CONN as follows: all normalization procedures are considered non-linear registration methods. (1) Realignment, (2) slice timing correction, (3) segmentation and normalization (non-linear transformation to Montreal neurological institute space), (4) scrubbing using the artifact detection tools with a threshold for global-signal above 5 (*z*-value) and subject-motion above 0.9 mm, and (5) smoothing using a 6 mm full-width half-maximum Gaussian kernel. After the preprocessing steps, a band-pass filter (0.008–0.09 Hz) was applied to the time series, and white matter and cerebrospinal fluid time series were regressed out. Group comparison in parameter for the head motion was performed and there was no significant group difference. The parcellation scheme of the Harvard-Oxford cortical and subcortical atlas determined 132 regions of interest (ROIs) (18). We conducted the whole-brain functional connectivity analysis (ROI-to-ROI) as described in our previous study with minor modification. The averaged time series for each ROI from the preprocessed images was extracted. Then, as a measure of functional connectivity, Pearson's correlation coefficient for each pair of the 132-time series was calculated and converted to *z*-scores using Fisher's *r*-to-*z* transformation. Then, we compared whole-brain functional connectivity between patients with BVP and control subjects with age and sex as covariates. We performed correlation analyses between functional connectivity and clinical variables (DHI, VADL, HADS, and disease duration) in patients with BVP with age and sex as covariates. We selected cluster-level inferences based on Functional Network Connectivity (FNC) multivariate parametric statistics with default settings in CONN toolbox (Functional Network Connectivity) (19). To control multiple comparison problems, we applied the statistical threshold at cluster-level false discovery rate-corrected two-sided *p*-values at *p* < 0.05 and a connection-level threshold of *p* < 0.05 uncorrected.

### Anatomical connectivity analysis

A total of 29 diffusion MRI scans (13 BVP subjects, 17 control subjects) were included in the connectometry database. Diffusion MRI connectometry was used to derive the correlational tractography that has fractional anisotropy (FA) values correlated with the group and clinical variables (DHI, VADL, HAD scores, and disease duration) (20). A nonparametric Spearman partial correlation was used to derive the correlation, and the effect of age and sex was removed using a multiple regression model. A total of 32 subjects were included in the analysis. A *T*-score threshold of 2.5 was assigned and tracked using a deterministic fiber tracking algorithm to obtain correlational tractography (21). A seeding region was placed at the whole brain. The tracks were filtered by topology-informed pruning with 4 iteration(s) (22). An FDR threshold of 0.05 was used to select tracks. To estimate the false discovery rate, a total of 4,000 randomized permutations were applied to the group label to obtain the null distribution of the track length.

### Voxel-based morphometry

The CAT12 toolbox version 1109 (http://dbm.neuro.unijena.de/cat/) was used to perform voxel-based morphometry. The T1weighted image was DARTEL-normalized to MNI space, segmented into gray matter, white matter, and cerebrospinal fluid, and smoothed with an 8-mm FWHM Gaussian kernel filter. The modulated normalized volume images were combined in a whole-brain voxel-wise statistical analysis (two-sample *t*-test) in the VBM approach. The age, sex, and total intracranial volume were entered as a covariate in the group comparison. DHI, VADL, HADS, and disease duration were tested for correlation with voxel-wise gray matter density in two separate random-effects multiple regression analyses. Results were thresholded at *p* < 0.001 (uncorrected) for cluster definition with a minimum cluster size of 100 voxels.

## Results

### Clinical features

The demographic and clinical characteristics and vestibular function tests of the BVP patients are presented in Table 2. Although the cause remains unclear in six BVP patients (46%), three patients had a history of general or local surgery and two patients had uncontrolled DM. seven patients (54%) of BVP had causative factors; three recurrent vestibular neuritis, two ototoxic drugs, one head trauma, and one SLE. All patients were not observed cerebellar dysfunction in neurologic examination and hearing abnormalities. Nine patients showed bilateral loss or reduction of low-frequency VOR (sum of bithermal max. peak SPV on each side < 6°/s by a caloric test with air stimulation) and five patients showed bilateral loss of high-frequency VOR (horizontal angular VOR gain < 0.6 by vHIT). The rotatory chair test was selectively performed when no clear abnormalities in the caloric test or vHIT. Nine of ten patients showed horizontal angular VOR gain lower than 0.1 upon sinusoidal stimulation (0.1 Hz, Vmax = 50°/s). The GDS score was significantly higher in BPV group (18.46 ± 7.42) than control group (4.53 ± 2.67, *p* = 0.004). Also, BVP group had a lower MMSE (27.46 ± 0.66) than the control group (29.82 ± 0.39, *p* < 0.001).

**Table 2:** Demography and vestibular function tests of patients with bilateral vestibulopathy.

**Patient**	**Gender**	**Age**	**Etiology**	**Duration (months)**	**Caloric test**						**vHIT**		**RC**	**DVA**	**DHI**	**VDRL**	**HADS**	**MMSE**	**GDS**
					**RW**	**RC**	**LW**	**LC**	**RT**	**LT**	**Rt VOR gain**	**Lt VOR gain**							
1	F	68	Idiopathic (severe DM)	240	0	0	0	0	0	0	0.65	0.6	0.01	+	78	131	–	24	28
2	M	62	Recurrent bilateral VN	300	0	2	0	2	2	2	0.93	0.25	0.06	−	4	27	8	29	5
3	F	49	Ototoxic (gentamicin)	3	4.4	3.6	2.2	6.3	8.0	8.5	0.72	0.52	0.1	−	46	99	19	30	29
4	F	59	Idiopathic (post-OP)	3	0	0	0	0	0	0	0.64	0.65	0.05	+	62	61	23	30	13
5	M	67	Recurrent bilateral VN	120	3	3	0	0	6.0	0	0.83	0.66	0.03	+	46	46	–	28	17
6	M	71	Idiopathic (post-OP)	4	0	0	1.9	1.2	0	3.1	0.57	0.52	0.04	+	56	69	25	29	24
7	M	63	Head trauma	22	3.8	2.4	4.4	4.8	6.2	9.2	0.71	0.56	0.1	−	50	42	20	29	24
8	F	67	Idiopathic (DM, CKD5)	18	0	0	0	0	0	0	0.35	0.36	0.04	+	76	141	23	24	23
9	F	22	SLE	3	1.4	5.9	6.9	3.2	7.3	10.1	0.34	0.39	–	+	94	152	16	30	19
10	M	80	Recurrent bilateral VN	4	1.7	0	1.1	0	1.7	1.1	0.65	0.34	0.2	+	62	134	17	28	15
11	M	75	AML, ototoxic	3	1.1	2	2.2	0	3.2	2.2	0.54	0.5	–	+	46	136	9	25	8
12	F	68	Idiopathic	180	0.6	0	0	0.4	0.6	0.4	0.72	0.53	–	+	54	53	24	27	22
13	F	74	Idiopathic (post-OP)	36	3.4	1.3	1.7	2.3	4.7	4.0	0.48	0.54	0.04	+	82	114	18	24	13
Mean		63.46		72.00	1.49	1.55	1.57	1.55	2.59	3.12	0.63	0.50			58.15	92.69		27.46	18.46
SD		14.70		103.755	1.63	1.84	2.09	2.09	3.00	3.74	0.17	0.12			22.47	44.34		2.40	7.42
Range (min–max)		22–80		3–300	0–4.40	0–5.9	0–6.9	0–6.3	0–8.0	0–10.1	0.34–0.93	0.25–0.66			4–94	27–152		24–30	5–28

RW, right warm stimulation; RC, right cold stimulation; LW, left warm stimulation; LC, left cold stimulation; RT, right total summation (RW + RC); LT, left total summation (LW + LC); vHIT, video-head impulse test; VOR, vestibulo-ocular reflex; RC, rotatory chair test; DVA, dynamic visual acuity; DHI, dizziness handicap inventory score; VDRL, vestibular disorders activities of daily living scale; HADS, hospital anxiety and depression scale; DM, diabetes mellitus; VN, vestibular neuritis; OP, operation; CKD, stage 5 chronic kidney disease; SLE, systemic lupus erythematosus; AML, acute myeloid leukemia; MMSE, mini-mental state examination; GDS, geriatric depression scale.

### Altered functional connectivity in patients with BVP

**Figure 1:**
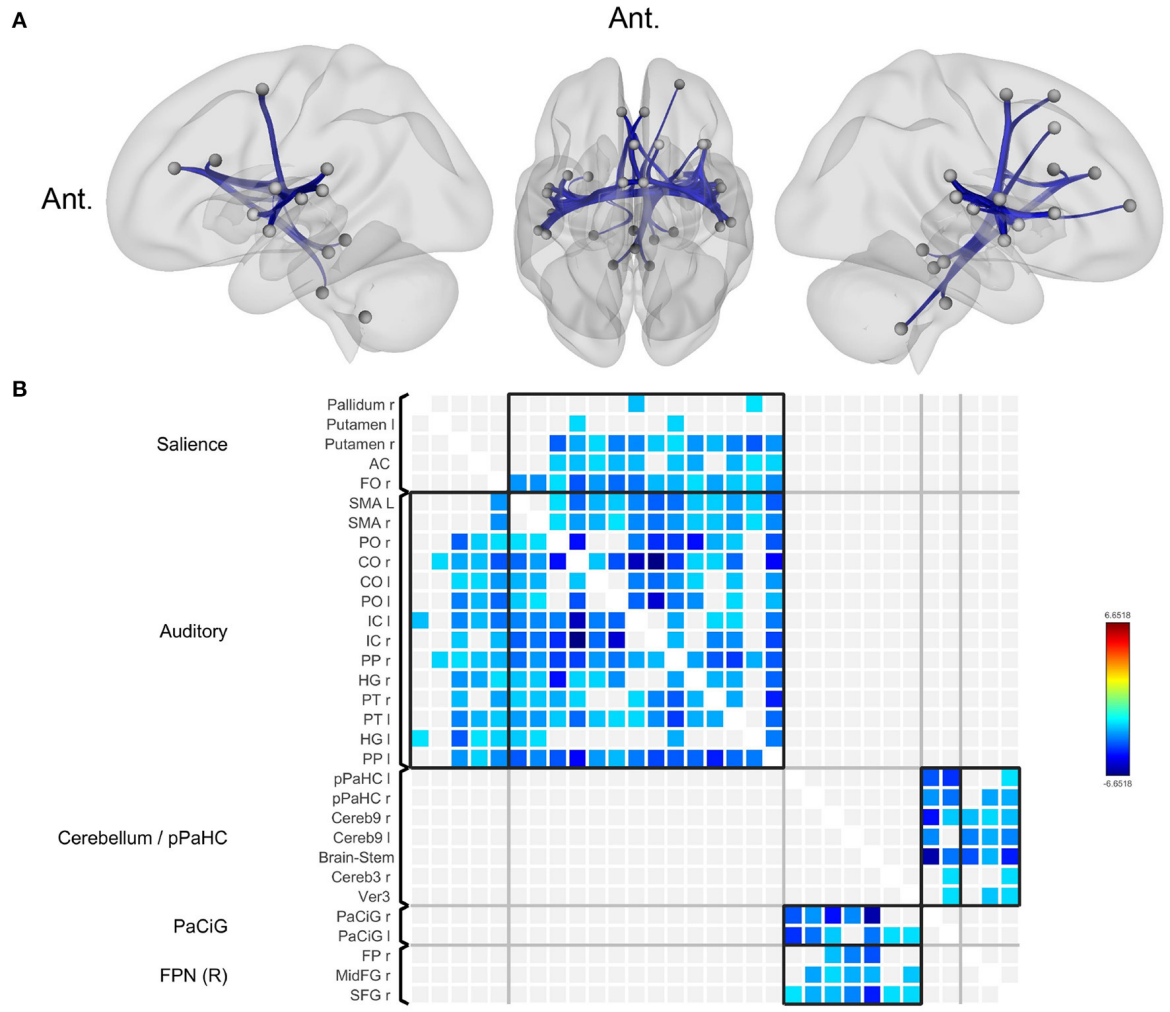
Decreased functional connectivity in bilateral vestibulopathy. **(A)** Patient with bilateral vestibulopathy showed decreased functional connectivity in the bilateral insular, lateral occipital, fusiform, and anterior cingulate cortices. Seed-level two-sided false discovery rate (FDR) corrected *p* < 0.05. **(B)** The connectivity matrix showed brain regions with reduced functional connectivity correspond to salience and auditory network.

Compared to normal controls, patients with BVP showed decreased functional connectivity among the key nodes of the salience network, auditory (including vestibular) network, bilateral posterior parahippocampal gyri, bilateral paracingulate gyri, and right frontoparietal network (Figure 1, Supplementary Table). Correlation analyses for clinical variables (DHI, VADL, HADS, and disease duration) in patients with BVP showed no significant result.

### Altered anatomical connectivity in patients with BVP

**Figure 2:**
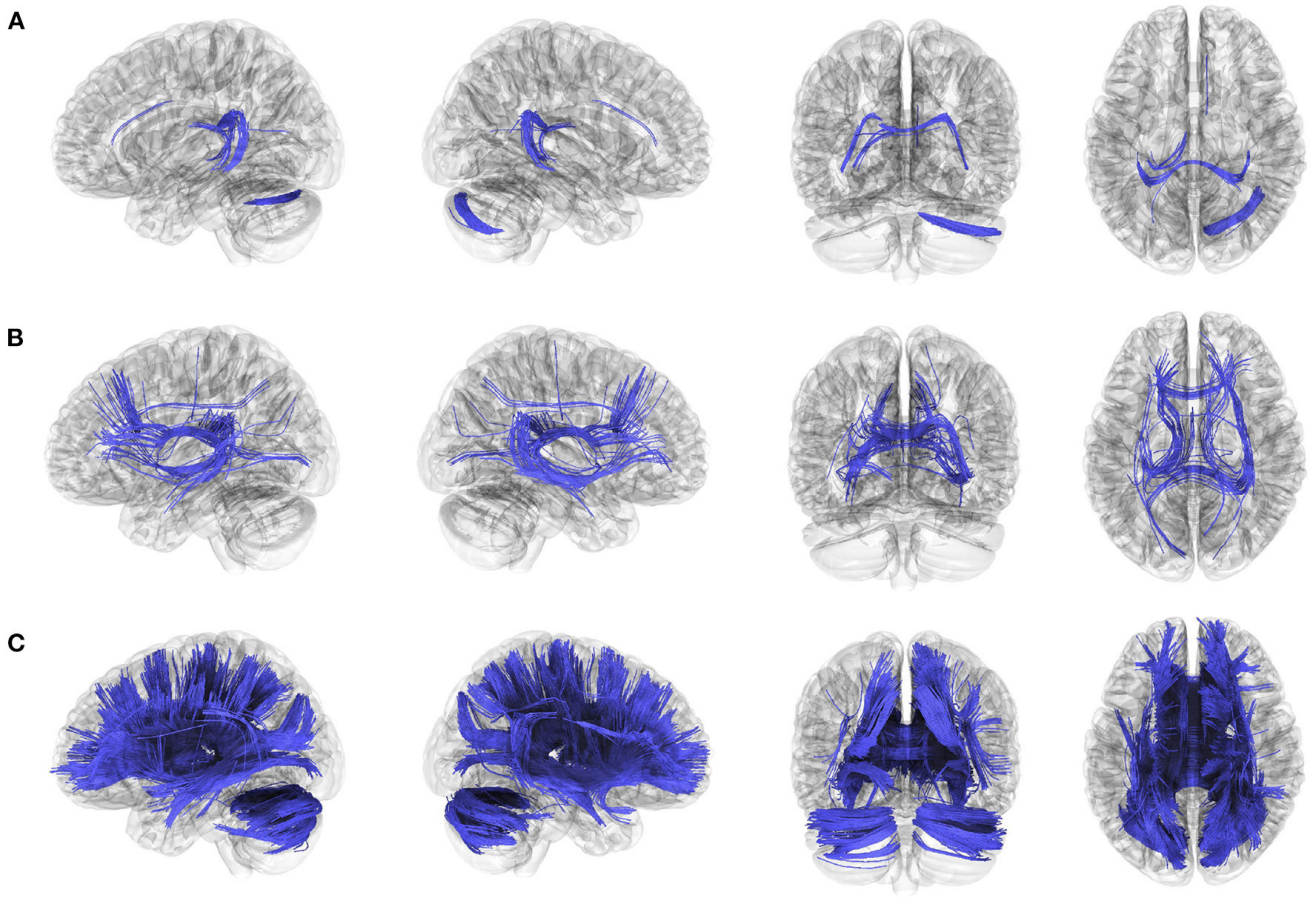
Decreased anatomical connectivity in bilateral vestibulopathy. **(A)** The anatomical connectometry analysis found fractional anisotropy (FA) values in the right cerebellum, corpus callosum tapetum, and left fornix decreased in the patients with bilateral vestibulopathy compared with control subjects (FDR ≤ 0.05). **(B)** The correlation analysis found FA values in the bilateral frontal parahippocampal cingulum, corpus callosum (tapetum, body, and forceps major), right inferior fronto-occipital fasciculus, bilateral fornix, negatively correlated with DHI scores in patients with bilateral vestibulopathy (FDR ≤ 0.05). **(C)** The correlation analysis found FA values in the corpus callosum (tapetum, body, forceps major, and forceps minor), bilateral fornix, bilateral cerebellum, bilateral superior and anterior thalamic radiation, right inferior fronto-occipital fasciculus, bilateral fronto-parietal cingulum, right dentatoruburothalamic tract negatively correlated with VADL scores in patients with bilateral vestibulopathy (FDR ≤ 0.05).

The anatomical connectometry analysis found fractional anisotropy (FA) values in the right cerebellum, corpus callosum tapetum, and left fornix decreased in the patients with BVP compared with control subjects (Figure 2A). The correlation analysis found FA values in the bilateral frontal parahippocampal cingulum, corpus callosum (tapetum, body, and forceps major), right inferior fronto-occipital fasciculus, bilateral fornix, negatively correlated with DHI in patients with BVP (Figure 2B). The correlation analysis found FA values in the corpus callosum (tapetum, body, forceps major, and forceps minor), bilateral fornix, bilateral cerebellum, bilateral superior and anterior thalamic radiation, right inferior fronto-occipital fasciculus, bilateral fronto-parietal cingulum, right dentatoruburothalamic tract negatively correlated with VADL in patients with BVP (Figure 2C).

### Voxel- and surface-based morphometry

**Figure 3:**
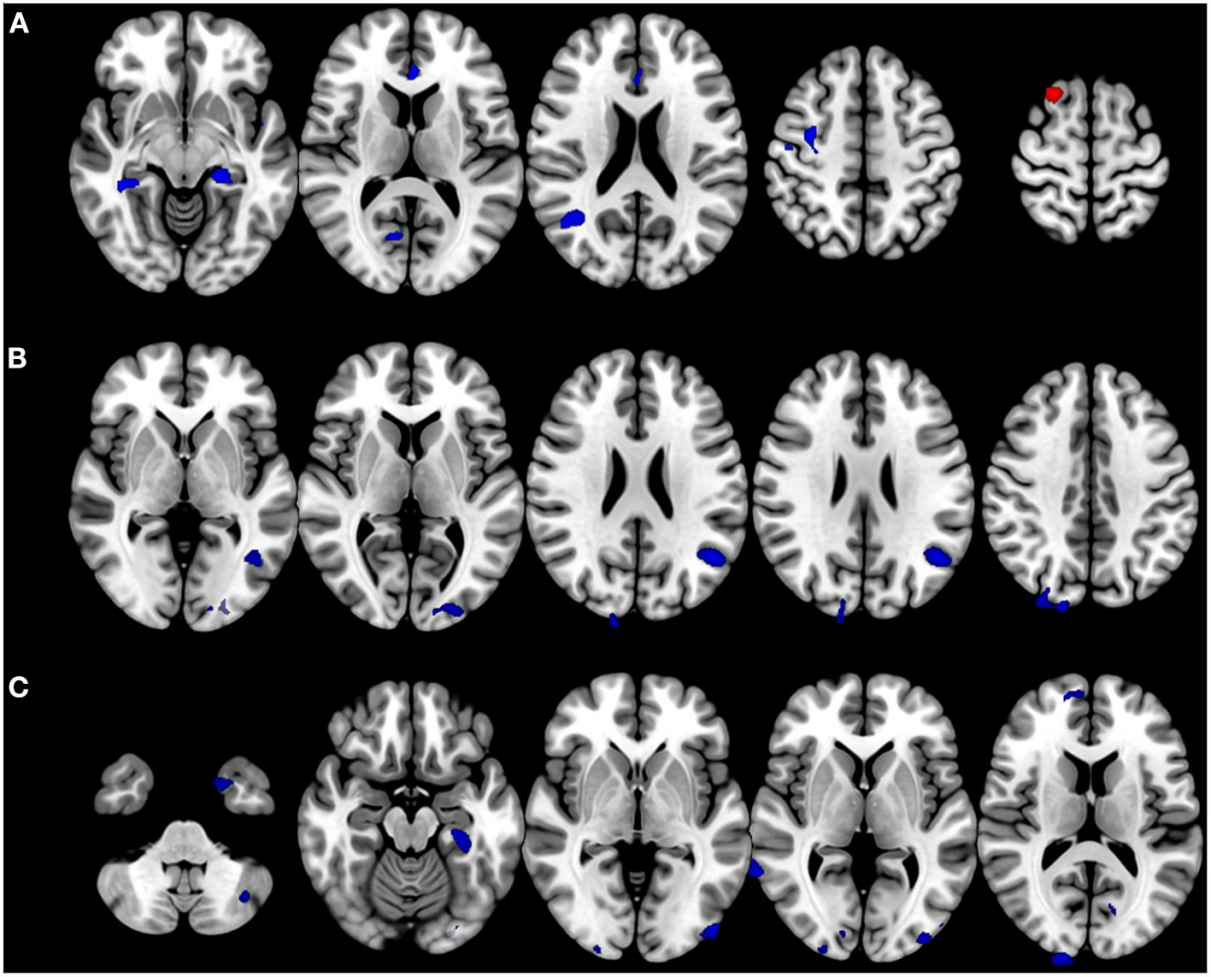
Alteration in brain volumes in bilateral vestibulopathy. **(A)** Patient with bilateral vestibulopathy showed decreased volumes in the bilateral parahippocampal gyri, right precentral gyrus, anterior cingulate gyrus, and right middle temporal gyrus. Gray matter volume in the right superior frontal gyrus increased in patients with bilateral vestibulopathy. **(B)** The correlation analysis found gray matter volumes in the bilateral middle occipital gyri, right superior occipital gyrus, left angular gyrus, and right cuneus negatively correlated with DHI scores in patients with bilateral vestibulopathy (uncorrected *p* ≤ 0.001, cluster extents > 100 voxels). **(C)** The correlation analysis found gray matter volumes in the right angular gyri, bilateral parahippocampal gyri, right middle temporal gyrus, right cuneus, bilateral inferior occipital gyri, left middle occipital gyrus, right superior frontal gyrus, left fusiform gyrus, bilateral caudate, left cerebellar crus, and bilateral calcarine gyri negatively correlated with VADL scores in patients with bilateral vestibulopathy (uncorrected *p* ≤ 0.001, cluster extents > 100 voxels).

Patients with BVP showed decreased volumes in the bilateral parahippocampal gyri, right precentral gyrus, anterior cingulate gyrus, and right middle temporal gyrus. Gray matter volume in the right superior frontal gyrus increased in the patients with BVP (Figure 3A). The correlation analysis found gray matter volumes in the bilateral middle occipital gyri, right superior occipital gyrus, left angular gyrus, and right cuneus negatively correlated with DHI in patients with BVP (Figure 3B). The correlation analysis found gray matter volumes in the right angular gyri, bilateral parahippocampal gyri, right middle temporal gyrus, right cuneus, bilateral inferior occipital gyri, left middle occipital gyrus, right superior frontal gyrus, left fusiform gyrus, bilateral caudate, left cerebellar crus, and bilateral calcarine gyri negatively correlated with VADL in patients with BVP (Figure 3C).

## Discussion

In our study, patients with BVP showed decreased functional connectivity among the key nodes of the salience network, auditory (including vestibular) network, bilateral posterior parahippocampal gyri, bilateral paracingulate gyri, and right frontoparietal network. The study also found decreased fractional anisotropy (FA) values in the right cerebellum, corpus callosum tapetum, and left fornix in patients with BVP compared with control subjects, and FA values in these regions were negatively correlated with DHI and VADL. Also, patients with BVP showed decreased volumes in the bilateral parahippocampal gyri, right precentral gyrus, anterior cingulate gyrus, and right middle temporal gyrus and these volume changes were negatively correlated with DHI and VADL.

Previous literature has reported conflicting results on the relationship between BVP and the volume of hippocampal formation. Some studies have found reduced volume in BVP patients (6, 8, 23), while others have not (24–26). Our study showed decreased volumes in the bilateral parahippocampal gyri. The parahippocampal cortex encompasses a large portion of the medial temporal lobe and is associated with many cognitive processes, including visuospatial processing and episodic memory (27). The present study provides evidence that supports the theory that the disruption of vestibular signals in BVP leads to the deafferentation of fibers that connect to the hippocampus, which in turn results in the atrophy of the hippocampus. This study offers a valuable perspective on the neural mechanisms underlying the symptoms exhibited by patients with BVP, including not only oscillopsia and imbalance but also deficiencies in spatial cognition (6, 8, 28).

The most noteworthy outcome of this study is the identification of a significant reduction in functional connectivity in several key regions, particularly the anterior cingulate and insular areas, which are known to be crucial nodes in the salience network (SN). The SN plays an important role in detecting and reacting to salient events, as well as facilitating access to attention and working memory resources. This interpretation is consistent with the findings of prior studies which have reported dysfunction of the SN in bilateral hearing loss (29, 30). Auditory as well as vestibular stimulation, is a crucial external stimulus for the detection of salient events. The study suggests that dysfunction of the salience network may be a common mediator of cognitive dysfunction that occurs as a result of abnormalities in peripheral sensory organs, such as hearing loss, not just in vestibular dysfunction.

SN is involved in switching between large-scale brain networks related to externally oriented attention and internally oriented mental processes. The SN, along with the central executive network, typically shows increased activation during cognitively demanding tasks, while the default-mode network shows decreased activation. When a salient event is detected, the SN initiates control signals to engage cognitive and task control systems and suppress the default-mode network (31). The SN is a causal hub for signaling the central executive network, which is important for maintaining and manipulating information in working memory (32). Therefore, dysfunction of the SN may also lead to dysfunction of the central executive network and the default mode network, which could potentially result in cognitive impairments, including attention, working memory, and spatial navigation, in patients with BVP.

The right superior frontal gyrus is one of the important nodes in the fronto-parietal network and is known to be involved in processing visual attention (33, 34). In BVP patients, loss of vestibular function results in increased dependence on visual stimulation. As a result of this compensatory action, it can be carefully deduced that the brain volume of the corresponding area increased.

The anatomical connectivity analysis demonstrated that there was a decrease in connectivity in various white matter tracts, which indicates that there are both functional and structural changes in the brain of patients with BVP. In addition, the correlation analysis showed that the more severe the impairment of daily life due to dizziness, the lower the integrity of the extensive white matter tract. This is presumed to be because, unlike the DHI, which consists of three domains, physical, emotional, and functional, VADL is less affected by other confounding effects such as anxiety or depression. After all, it is a scale focused only on disturbances in daily life due to balance disorders.

There are severe limitations to our study. First, the sample size was small, which did not allow further subgroup analysis of etiologies. Second, etiology of BVP or result of vestibular function test are heterogenous. Third, we excluded patients with MMSE score ≤ 23 to rule out the dementia, but the BVP group had a lower MMSE (27.46 ± 0.66) than the control group (29.82 ± 0.39). Fourth, we excluded psychiatric disorder such as generalized anxiety disorder or major depressive disorder, but the GDS and HADS scores in the BVP group were higher than in the control group, which may have affected the results.

Despite its limitations, this study has the strength of being a comprehensive and data-driven examination of structural and functional brain imaging of BVP.

## Conclusion

Our study concurs with previously established characteristics of BVP, such as reductions in the volume of the hippocampus and alterations in functional and anatomical connectivity. Furthermore, the results of this study propose that dysfunction in the salience network may serve as a common mediator for cognitive dysfunction that arises from abnormalities in peripheral sensory organs.

## Data availability statement

The raw data supporting the conclusions of this article will be made available by the authors, without undue reservation.

## Ethics statement

The studies involving human participants were reviewed and approved by Institutional Review Board of Ulsan University Hospital (IRB no. 2018-06-016). The patients/participants provided their written informed consent to participate in this study.

## Author contributions

J-YP, E-SL, and J-SK conceived and planned the research. E-SL and YW contributed to analyzing the image data. J-YP and E-SL took the lead in writing the manuscript. All authors provided critical feedback and helped shape the research, analysis, and manuscript. All authors contributed to the article and approved the submitted version.

## References

[1] Baloh RW Jacobson K Honrubia V. Idiopathic bilateral vestibulopathy. Neurology. (1989) 39:272–5. 10.1212/WNL.39.2.272 2783767

[2] Fujimoto C Yagi M Murofushi T. Recent advances in idiopathic bilateral vestibulopathy: a literature review. Orphanet J Rare Dis. (2019) 14:202. 10.1186/s13023-019-1180-8 31426838 PMC6701126

[3] Lucieer F Duijn S Van Rompaey V Perez Fornos A Guinand N Guyot JP. Full spectrum of reported symptoms of bilateral vestibulopathy needs further investigation-a systematic review. Front Neurol. (2018) 9:352. 10.3389/fneur.2018.00352 29915554 PMC5994412

[4] Bessot N Denise P Toupet M Van Nechel C Chavoix C. Interference between walking and a cognitive task is increased in patients with bilateral vestibular loss. Gait Posture. (2012) 36:319–21. 10.1016/j.gaitpost.2012.02.021 22465706

[5] Popp P Wulff M Finke K Ruhl M Brandt T Dieterich M. Cognitive deficits in patients with a chronic vestibular failure. J Neurol. (2017) 264:554–63. 10.1007/s00415-016-8386-7 28074268

[6] Kremmyda O Hüfner K Flanagin VL Hamilton DA Linn J Strupp M. Beyond dizziness: virtual navigation, spatial anxiety and hippocampal volume in bilateral vestibulopathy. Front Hum Neurosci. (2016) 10:139. 10.3389/fnhum.2016.00139 27065838 PMC4814552

[7] Dobbels B Mertens G Gilles A Claes A Moyaert J van de Berg R. Cognitive function in acquired bilateral vestibulopathy: a cross-sectional study on cognition, hearing, and vestibular loss. Front Neurosci. (2019) 13:340. 10.3389/fnins.2019.00340 31105513 PMC6492511

[8] Brandt T Schautzer F Hamilton DA Brüning R Markowitsch HJ Kalla R. Vestibular loss causes hippocampal atrophy and impaired spatial memory in humans. Brain. (2005) 128:2732–41. 10.1093/brain/awh617 16141283

[9] Grabherr L Cuffel C Guyot JP Mast FW. Mental transformation abilities in patients with unilateral and bilateral vestibular loss. Exp Brain Res. (2011) 209:205–14. 10.1007/s00221-011-2535-0 21287158

[10] Dobbels B Peetermans O Boon B Mertens G Van de Heyning P Van Rompaey V. Impact of bilateral vestibulopathy on spatial and nonspatial cognition: a systematic review. Ear Hear. (2019) 40:757–65. 10.1097/AUD.0000000000000679 31242136

[11] Bense S Deutschländer A Stephan T Bartenstein P Schwaiger M Brandt T. Preserved visual-vestibular interaction in patients with bilateral vestibular failure. Neurology. (2004) 63:122–8. 10.1212/01.WNL.0000129545.79566.6A 15249621

[12] Göttlich M Jandl NM Wojak JF Sprenger A von der Gablentz J Münte TF. Altered resting-state functional connectivity in patients with chronic bilateral vestibular failure. Neuroimage Clin. (2014) 4:488–99. 10.1016/j.nicl.2014.03.003 24818075 PMC3984447

[13] Strupp M Kim JS Murofushi T Straumann D Jen JC Rosengren SM. Bilateral vestibulopathy: diagnostic criteria consensus document of the classification committee of the bárány society. J Vestib Res. (2017) 27:177–89. 10.3233/VES-170619 29081426 PMC9249284

[14] Oldfield RC. The assessment and analysis of handedness: the Edinburgh inventory. Neuropsychologia. (1971) 9:97–113. 10.1016/0028-3932(71)90067-4 5146491

[15] Han GC Lee EJ Lee JH Park SN Lee HY Jeon EJ. The study of standardization for a Korean adaptation of self-report measures of dizziness. J Korean Bal Soc. (2004) 3:307–25.

[16] Zigmond AS Snaith RP. The hospital anxiety and depression scale. Acta Psych Scand. (1983) 67:361–70. 10.1111/j.1600-0447.1983.tb09716.x 6880820

[17] Shepard NT Jacobson GP. The caloric irrigation test. Handb Clin Neurol. (2016) 137:119–31. 10.1016/B978-0-444-63437-5.00009-1 27638067

[18] Caviness VS Kennedy DN Richelme C Rademacher J Filipek PA. The human brain age 7–11 years: a volumetric analysis based on magnetic resonance images. Cereb Cortex. (1996) 6:726–36. 10.1093/cercor/6.5.726 8921207

[19] Jafri MJ Pearlson GD Stevens M Calhoun VD. A method for functional network connectivity among spatially independent resting-state components in schizophrenia. Neuroimage. (2008) 39:1666–81. 10.1016/j.neuroimage.2007.11.001 18082428 PMC3164840

[20] Yeh FC Badre D Verstynen T. Connectometry: a statistical approach harnessing the analytical potential of the local connectome. Neuroimage. (2016) 125:162–71. 10.1016/j.neuroimage.2015.10.053 26499808

[21] Yeh FC Verstynen TD Wang Y Fernández-Miranda JC Tseng WY. Deterministic diffusion fiber tracking improved by quantitative anisotropy. PLoS ONE. (2013) 8:e80713. 10.1371/journal.pone.0080713 24348913 PMC3858183

[22] Yeh FC Panesar S Barrios J Fernandes D Abhinav K Meola A. Automatic removal of false connections in diffusion MRI tractography using topology-informed pruning (TIP). Neurotherapeutics. (2019) 16:52–8. 10.1007/s13311-018-0663-y 30218214 PMC6361061

[23] Hufner K Strupp M Smith P Brandt T Jahn K. Spatial separation of visual and vestibular processing in the human hippocampal formation. Ann N Y Acad Sci. (2011) 1233:177–86. 10.1111/j.1749-6632.2011.06115.x 21950991

[24] Göttlich M Jandl NM Sprenger A Wojak JF Münte TF Krämer UM. Hippocampal gray matter volume in bilateral vestibular failure. Hum Brain Mapp. (2016) 37:1998–2006. 10.1002/hbm.23152 26918638 PMC6867258

[25] Cutfield NJ Scott G Waldman AD Sharp DJ Bronstein AM. Visual and proprioceptive interaction in patients with bilateral vestibular loss. Neuroimage Clin. (2014) 4:274–82. 10.1016/j.nicl.2013.12.013 25061564 PMC4107374

[26] Van Cruijsen N Hiemstra WM Meiners LC Wit HP Albers FW. Hippocampal volume measurement in patients with Ménière's disease: a pilot study. Acta Otolaryngol. (2007) 127:1018–23. 10.1080/00016480601127000 17851902

[27] Aminoff EM Kveraga K Bar M. The role of the parahippocampal cortex in cognition. Trends Cogn Sci. (2013) 17:379–90. 10.1016/j.tics.2013.06.009 23850264 PMC3786097

[28] Smith PF. The vestibular system and cognition. Curr Opin Neurol. (2017) 30:84–9. 10.1097/WCO.0000000000000403 27845944

[29] Xu XM Jiao Y Tang TY Lu CQ Zhang J Salvi R. Altered spatial and temporal brain connectivity in the salience network of sensorineural hearing loss and tinnitus. Front Neurosci. (2019) 13:246. 10.3389/fnins.2019.00246 30941010 PMC6433888

[30] Li Z Zhu Q Geng Z Song Z Wang L Wang Y. Study of functional connectivity in patients with sensorineural hearing loss by using resting-state fMRI. Int J Clin Exp Med. (2015) 8:569–78. 25785031 PMC4358486

[31] Seeley WW. The salience network: a neural system for perceiving and responding to homeostatic demands. J Neurosci. (2019) 39:9878–82. 10.1523/JNEUROSCI.1138-17.2019 31676604 PMC6978945

[32] Engström M Landtblom AM Karlsson T. Brain and effort: brain activation and effort-related working memory in healthy participants and patients with working memory deficits. Front Hum Neurosci. (2013) 7:140. 10.3389/fnhum.2013.00140 23616756 PMC3628360

[33] Coull JT Frith CD Frackowiak RS Grasby PM A. fronto-parietal network for rapid visual information processing: a PET study of sustained attention and working memory. Neuropsychologia. (1996) 34:1085–95. 10.1016/0028-3932(96)00029-2 8904746

[34] Taran N Farah R DiFrancesco M Altaye M Vannest J Holland S. The role of visual attention in dyslexia: Behavioral and neurobiological evidence. Hum Brain Mapp. (2022) 43:1720–37. 10.1002/hbm.25753 34981603 PMC8886655

